# Major Disasters’ Impacts on Long-Term Care Settings, Vulnerable Older Adults, and Care Providers

**DOI:** 10.1093/geroni/igab046.770

**Published:** 2021-12-17

**Authors:** Leah Haverhals, Katie Cherry

## Abstract

The COVID-19 pandemic has disproportionately negatively affected older adults, and has specifically devasted older adults who are minorities and those who reside in long-term care (LTC) facilities. For professionals working in LTC facilities, major stressors and challenges due to the pandemic must be navigated, sometimes in parallel with the effect that major disasters like hurricanes can have on LTC facilities. This symposium will focus on the impact major disasters, including the COVID-19 pandemic and Hurricane Irma, had on LTC settings and those who live and work there, as well as older adults who are minorities and their communities. First, Dr. Roma Hanks will present findings from a study of community members and leaders in a majority African-American community in the United States (US) about their experiences with and challenges faced related to the pandemic. Second, Dr. Lisa Brown will share experiences and perceptions of mental health clinicians from across the US who worked in LTC settings before and during the pandemic. Third, Dr. Ella Cohn-Schwartz will describe how the pandemic impacted Holocaust survivors ages 75+ in Israel compared to older adults who did not experience the Holocaust. Fourth, Dr. Lindsay Peterson will present findings from interviews with nursing home and assisted living community representatives in the US regarding vulnerabilities LTC facilities experienced related to Hurricane Irma in 2017. As a whole, these presenters will provide insights into experiences of older adults, care providers, LTC facilities, and communities as they navigated challenges associated with the COVID-19 pandemic and a major hurricane.

